# Effectiveness of the WHO Safe Childbirth Checklist program in reducing severe maternal, fetal, and newborn harm in Uttar Pradesh, India: study protocol for a matched-pair, cluster-randomized controlled trial

**DOI:** 10.1186/s13063-016-1673-x

**Published:** 2016-12-07

**Authors:** Katherine E. A. Semrau, Lisa R. Hirschhorn, Bhala Kodkany, Jonathan M. Spector, Danielle E. Tuller, Gary King, Stuart Lipsitz, Narender Sharma, Vinay Pratap Singh, Bharath Kumar, Neelam Dhingra-Kumar, Rebecca Firestone, Vishwajeet Kumar, Atul A. Gawande

**Affiliations:** 1 Ariadne Labs/Harvard Medical School, 401 Park Drive, 3rd floor East, Boston, MA 02115 USA; 2 JNMC Medical College, Belgaum, 590010 Karnataka India; 3 Novartis Institutes for BioMedical Research, 250 Massachusetts Avenue, Cambridge, MA 02138 USA; 4 Ariadne Labs/Harvard T.H. Chan School of Public Health, 401 Park Drive, 3rd floor East, Boston, MA 02115 USA; 5 Harvard University, 1737 Cambridge Street Harvard University, Cambridge, MA 02138 USA; 6 Brigham and Women’s Hospital, 1620 Tremont Street, 4-020, Boston, MA 02120 USA; 7 Population Services International, 2/325, Vijayant Khand, Gomti Nagar, Lucknow, 226010 Uttar Pradesh India; 8 World Health Organization, 20, Avenue Appia, 1211, Geneva 27, Switzerland; 9 Population Services International, 1120 19th Street, NW, Suite 600, Washington, DC 20036 USA; 10 Community Empowerment Lab, 26/11 Wazeer Hassan Road, Lucknow, 226001 India; 11 Ariadne Labs/Harvard T.H. Chan School of Public Health, 677 Huntington Avenue, Boston, MA 02115 USA

**Keywords:** Cluster-randomized controlled trial, WHO Safe Childbirth Checklist, Maternal health, Newborn health, Perinatal health, Stillbirth, Coaching, Supportive supervision, Quality improvement, India

## Abstract

**Background:**

Effective, scalable strategies to improve maternal, fetal, and newborn health and reduce preventable morbidity and mortality are urgently needed in low- and middle-income countries. Building on the successes of previous checklist-based programs, the World Health Organization (WHO) and partners led the development of the Safe Childbirth Checklist (SCC), a 28-item list of evidence-based practices linked with improved maternal and newborn outcomes. Pilot-testing of the Checklist in Southern India demonstrated dramatic improvements in adherence by health workers to essential childbirth-related practices (EBPs). The BetterBirth Trial seeks to measure the effectiveness of SCC impact on EBPs, deaths, and complications at a larger scale.

**Methods/design:**

This matched-pair, cluster-randomized controlled, adaptive trial will be conducted in 120 facilities across 24 districts in Uttar Pradesh, India. Study sites, identified according to predefined eligibility criteria, were matched by measured covariates before randomization. The intervention, the SCC embedded in a quality improvement program, consists of leadership engagement, a 2-day educational launch of the SCC, and support through placement of a trained peer “coach” to provide supportive supervision and real-time data feedback over an 8-month period with decreasing intensity. A facility-based childbirth quality coordinator is trained and supported to drive sustained behavior change after the BetterBirth team leaves the facility.

Study participants are birth attendants and women and their newborns who present to the study facilities for childbirth at 60 intervention and 60 control sites. The primary outcome is a composite measure including maternal death, maternal severe morbidity, stillbirth, and newborn death, occurring within 7 days after birth. The sample size (*n* = 171,964) was calculated to detect a 15% reduction in the primary outcome. Adherence by health workers to EBPs will be measured in a subset of births (*n* = 6000).

The trial will be conducted in close collaboration with key partners including the Governments of India and Uttar Pradesh, the World Health Organization, an expert Scientific Advisory Committee, an experienced local implementing organization (Population Services International, PSI), and frontline facility leaders and workers.

**Discussion:**

If effective, the WHO Safe Childbirth Checklist program could be a powerful health facility-strengthening intervention to improve quality of care and reduce preventable harm to women and newborns, with millions of potential beneficiaries.

**Trial registration:**

BetterBirth Study Protocol dated: 13 February 2014; ClinicalTrials.gov: NCT02148952; Universal Trial Number: U1111-1131-5647.

**Electronic supplementary material:**

The online version of this article (doi:10.1186/s13063-016-1673-x) contains supplementary material, which is available to authorized users.

## Background

Maternal and neonatal morbidity and mortality remain unacceptably high, despite a focus on improving the health of pregnant women and newborns through Millennium Development Goals 4 and 5 [1–3]. Gaps in care during labor, delivery, and the early neonatal period are well-recognized, yet few simple and scalable strategies have proven to be effective to support health worker adherence to clinical, essential childbirth-related practices (EBPs). Poor quality of care is of particular concern in low- and middle-income countries where the majority of avoidable maternal, fetal, and newborn morbidity and mortality occurs [1, 2, 4].

One current global strategy is to shift childbirth from the home to facilities, but evidence suggests that facilities in many high-risk areas may be ill-prepared to safely care for the increased patient load that this would generate. The example of India is illustrative. As part of the National Health Mission’s (NHM) Reproductive and Child Health Program, the Government of India (GoI) launched in 2005 the Janani Suraksha Yojana (JSY), a scheme to promote institutional deliveries by linking cash incentives for patients and Accredited Social Health Activist (ASHA) workers with delivery and post-delivery care at the facility. Cost reimbursement is also provided for transport to and from health facilities [5]. From 2005 to 2010, JSY has resulted in a marked increase in the number of institutional deliveries (25% to 53.3%), but poor quality of care has limited the potential benefits of facility-based delivery to these women and their neonates [6]. Identifying and instituting effective, scalable interventions at health facilities is critical to improving quality and safety of childbirth.

Checklist-based programs have been used with increasing spread and effect in health care to help providers translate evidence into high-quality patient care [7]. This approach to improve quality has been shown to significantly improve health outcomes in intensive care medicine and surgery, including in low-resource settings [8, 9]. To translate this approach to reduce maternal and newborn harm, a partnership between the World Health Organization (WHO) and more than 100 frontline health workers and technical experts was established to develop a safe childbirth checklist program. The WHO Safe Childbirth Checklist (SCC) is comprised of 29 evidence-based practices that are associated with improved maternal and newborn outcomes. Over a 2-year period, the SCC was developed through a systematic process including evidence and guideline review, extensive consultation with content experts and potential users, and field evaluation for usability in Africa, Asia, and the Middle East [10]. Checklist items address the major causes of maternal and newborn mortality in resource-limited settings. The SCC is focused on care delivered in facilities for births at term gestation as term delivery represents the overwhelming majority of births. The Checklist is designed to address quality of care at four critical periods in the birth continuum: on admission to facility, at the time of pushing (or before cesarean delivery), soon after birth (within 1 h), and at discharge (see Additional file 1: Figure S1).

Limited data are available on the impact of SCC-based programs on the uptake of EBPs and no published studies have examined the impact on maternal and infant mortality and morbidity [11, 12]. In 2010, the SCC program was pilot-tested at a public-sector, subdistrict-level hospital in Karnataka, India [11]. Through a pre-post intervention design, the program’s impact on health workers’ adherence to EBPs was assessed. The SCC program resulted in dramatic improvements in adherence (increase from mean of 10/29 (34%) to 25/29 EBPs (86%), *p* < 0.001). While the pilot study demonstrated that the SCC program improved health worker performance, the study was not powered to detect a change in maternal and perinatal health outcomes. In Sri Lanka, the results of SCC program implementation at a tertiary-level hospital were assessed through surveys of birth attendants, who reported an average of 21 out of 29 EBPs conducted around the time of each delivery [12]. While adherence to the Checklist is important, understanding the impact on maternal/neonatal harm is essential for providing evidence to support broad-scale expansion of the SCC-based programs.

The BetterBirth Trial will measure the impact that the BetterBirth approach (checklist + coaching + data feedback) has on deaths and complications in institutional childbirths through an adaptive, matched-pair, cluster-randomized controlled study. The study will take place in the state of Uttar Pradesh (UP), India, a largely rural state with among the highest rates of maternal and newborn mortality in India and globally. It is anticipated that results from the BetterBirth Trial will be used to inform policy-making and program development to improve quality of care and clinical safety using the SCC for mothers and their newborns in India and other high-burden countries.

## Methods/design

### Trial design

Using a matched-pair, cluster-randomized controlled design, the intervention will be delivered at the facility (i.e., cluster) level with the primary outcome measured at the individual level (i.e., mother, fetus, or newborn). The SCC program will be introduced at 60 facilities (intervention arm); the other 60 facilities will receive standard of care services (control arm; 120 sites total). Facilities will be enrolled as a matched pair (one intervention and one control site) at the same time. All 60 pairs will be initiated in a staggered fashion over a 15-month period. In each matched pair, data collection for primary outcomes will initiate 7 − 10 weeks after coaching begins in the intervention and continue for up to 12 months.

Study sites will be identified from across UP, selected according to predefined eligibility criteria. Criteria include accessibility to investigators; a government-accredited health center at the primary (PHC), or community (CHC) level; three or more birth attendants on the staff who have training at the level of an auxiliary nurse midwife or higher; a birth volume of at least 1000 births annually; absence of ongoing research or other interventions that could confound trial results; and willingness of administrative and clinical leaders to participate. Only public sector health facilities will be enrolled.

Uttar Pradesh has 75 administrative districts. To maximize representation and to facilitate administrative processes, five geographic “hubs” spread across the state will be selected based on maximizing (1) rates of institutional births, (2) absence of concurrent institutional childbirth quality improvement programs, and (3) prioritization by the Government of UP. Each hub will be comprised of four to six districts with approximately 20 enrolled facilities per hub.

Matched-pairing of study sites before randomization will be conducted by coarsened exact matching, a monotonic imbalance bounding matching method that estimates causal effects by reducing imbalance in covariates between treated and comparison groups [13–15]. Matching will increase statistical power and efficiency, and robustness to experimental failures [14, 16, 17]. Facilities will be matched based on functional classification (PHC, CHC, or CHC/first referral unit (FRU)); geographic location (i.e., distance to district hospital (DH)); annual birth volume; and number of birth attendants. Within each matched pair of clusters, one site will be randomized to receive the intervention and the other will be the control site. Baseline facility data will be compared to assure that matching was successful. If a facility that is enrolled in the trial exits the trial after enrollment, the paired facility will also be dropped.

### Setting

Uttar Pradesh is India’s most populous state with more than 203 million people; 29.4% live below the poverty line and 77% live in rural areas [18, 19]. In UP, 773 CHC facilities and 3497 PHC facilities provide services to the population [20]. National initiatives aimed at improving maternal and newborn health in India, including JSY, have resulted in dramatic increase in rates of institutional delivery, from 17.5% in 2005 − 2006 to above 50% in 2008 [21]. However, high rates of maternal and neonatal mortality persist; the maternal mortality ratio is 258 deaths/100,000 live births and the neonatal mortality rate is 49 deaths/1000 live births in UP [22].

### Study population

Women presenting for childbirth at study facilities and their newborns will be enrolled, excluding those who have been referred into the facility by interfacility transfer and excluding women who are being managed for abortion. Women or newborns from enrolled sites who are referred out to another facility (before or after delivery) will remain in the study with their outcomes allocated to the referring facility.

Health care workers providing services during childbirth will also be enrolled if they consent for observation of EBPs and participation in Checklist acceptability and utilization surveys.

#### Study site enrollment

Approval and support will be obtained from district chief medical officers, after which facilities will be approached for participation. The program will be outlined to facility leadership including the head of the facility (i.e., medical officer-in-charge (MOIC) or medical superintendent (MS)) from whom a formal commitment to participating in the study will be obtained.

### Intervention

The SCC, a bedside tool, prompts health workers to conduct essential, evidence-based childbirth practices. The SCC is included in the Government of India Maternal and Neonatal Health Toolkit (http://nrhm.gov.in/images/pdf/programmes/maternal-health/guidelines/MNH_Toolkit_23_11_2013.pdf); however, it has not been used widely. The investigators worked with the GoI and UNICEF-India to align the SCC with national guidelines (Additional file 1: Figure S1). The adapted checklist used in the BetterBirth Trial has 28 items to be conducted at four junctures (“pause points”) in clinical care: on admission, at the time of pushing (or before cesarean delivery), soon after birth (within 1 h), and at discharge.

The BetterBirth approach is designed to maximize the likelihood of successful introduction of the Checklist into clinical practice and will be based on a model for facilitating change that consists of Engage, Launch, and Support based on five key messages (Table 1). Within each hub, an implementation team consisting of a senior coach manager (physician or public health practitioner with 8 − 10 years of experience), coach team leaders (a physician or trained public health practitioner with at least 4 years of experience) and nurse coaches trained in childbirth practices, SCC use and coaching techniques will provide support for facilities. Typically, a coach manages two to four facilities at any given time; a coach team leader can manage four to five facilities at one time.

#### Engage

At each facility, the team leader will engage the senior administrative and clinical leaders of the facility to discuss current childbirth-related maternal and child health issues and the potential applicability of the SCC to the facility.

#### Launch

The coach team leader and coach will provide a 2-day orientation to the SCC and quality of care at childbirth, using an implementation flipbook and conceptual videos to illustrate how the Checklist could be integrated into care and utilized correctly. Further, a gap analysis is conducted with facility staff to identify issues that deter staff from providing the best care.

#### Support

In each facility, a locally designated Childbirth Quality Coordinator (CQC) will be identified within the first 2 months and trained to help lead implementation and improvement efforts at that site. Following the launch, the team leader will continue to visit each facility regularly (initially weekly, then fortnightly, and finally monthly) to provide support to the CQC and administrative leaders including addressing system barriers to effective SCC use such as birth supplies and staff turnover. Over an 8-month period, the coach will visit each facility twice-weekly for the first 4 months, then weekly for 2 months, then every 2 weeks for a month, with one final visit in the 8th month. Coaches will observe the birth attendant, record the birth practices, and identify barriers that prevent implementation of best practices. Coaches diagnose behavioral barriers along with the birth attendants. Observations will be entered into a mobile application (app) and heat-maps of behavior over time will be automatically generated. At each visit, the coach will review progress, including review of completed SCCs since the prior coaching visit and heat-maps, and tailor the coaching to address gaps in clinical practices, overcome the identified barriers, or issues related to supplies, and will coach the birth attendants in effective checklist use. Checklist utilization will be monitored and followed up over time by the team leader, coach, and the facility-based CQC to ensure barriers to Checklist use, including supplies, are identified and addressed with the caregiver and district and facility leadership, if necessary. No clinical skill training and no additional commodities are supplied by the intervention team to the facility.

There will be no intervention initially in the comparison arm clusters apart from the introduction of a standardized birth register for data collection (see below). At the conclusion of the trial, components of the intervention will be rolled out to all facilities if the program is effective.

### Outcomes

The primary outcome is a summary composite measure of maternal death, maternal severe morbidity, intrapartum-related stillbirth, or newborn death occurring within 7 days after birth (see Table 1). A composite metric was selected because the SCC program is intended to reduce harm for both women and newborns, and is also meant to influence intrapartum management thereby reducing fetal harm. The primary outcome will be measured by examining each of the component outcomes separately and subsequently using a test with three degrees of freedom to assess the causal effects of the three outcomes on the composite measure. Outcomes occurring during delivery or within 7 days postpartum will be captured as the SCC program is anticipated to have beneficial effects that extend into the early postpartum period. In addition, most women and newborns in the trial setting leave the facility soon after birth (many in under 12 h) and hence a substantial number of very early outcomes would be missed if the data collection was limited to in-hospital occurrences.

**Table 1:** Five key messages of the SCC program

1. You can prevent complications: Most conditions leading to maternal and newborn deaths can be avoided by simple actions. The BetterBirth Checklist Program will help you overcome the challenges of providing consistently safe care during childbirth. 2. Pause for 4 Pause Points during childbirth care: There are 4 Pause Points during childbirth when it is important for you to be sure that the woman and baby are safe. 3. Perform Essential Actions at each Pause Points: At each Pause Point, you must complete a set of Essential Actions. 4. Prepare for routine care and possible crisis situations: Preparing for each Essential Action will help prevent crisis situations. Preparation occurs in advance. 5. Empower women and their companions: Women and their companions can help you to keep the woman and baby safe.	

Maternal severe morbidities are included in the composite to provide a measure of effect on maternal health, since the relative infrequency of maternal death is impractical to use as a sole indicator of maternal harm [23]. The WHO has published clinical, laboratory-based, and management-based criteria for defining maternal near-missed events (sometimes called “life-threatening conditions”) [24, 25]. In this trial, the WHO near-missed death criteria have been modified to contain a set of five clinical conditions that can be practically captured by self-report. The occurrence of any one of these five conditions will constitute a maternal severe morbidity. Management-based criteria including blood transfusion, hysterectomy and return to the health facility for a problem will not be included in the composite measure, but will be analyzed separately.

Prespecified secondary outcomes in the trial are listed in Table 2. Additional outcomes include the impact of the intervention on resource availability of essential childbirth commodities; Checklist utilization; cost-effectiveness; rates of medication administration; patient satisfaction with delivery experiences; family planning decisions; and health worker attitudes relating to safety of mothers and newborns (see Additional file 2 and Additional file 3: Figure S2).

**Table 2:** Primary and secondary trial outcomes

A. *Combined maternal, fetal and newborn outcome*: composite rate of maternal death by 7 days, intrapartum-related stillbirth, early neonatal death by 7 days, and maternal severe morbidity by 7 days. [Primary outcome] B. *Maternal outcomes:* Rate of maternal death (by 7 days), rate of maternal severe morbidity (by 7 days), rate of inter-facility transfer, blood transfusion, hysterectomy, facility revisit, and C-section rate. C. *Newborn outcomes:* Rate of intrapartum-related stillbirth, rate of early neonatal death (by 7 days), facility revisit, and rate of inter-facility transfer. *D. Rates of adherence by health workers to essential childbirth practices:* In a sample of total births, uptake of the following practices will be assessed: maternal temperature obtained on admission, maternal blood pressure obtained on admission, partograph use, appropriate hand hygiene (use of soap and water, and wearing clean gloves) by health workers before 1^st^ vaginal examination, oxytocin administration within 1 minute after birth, appropriate intervention for the newborn if apneic at birth, newborn weight and temperature obtained within 1 hour after birth, and initiation of breastfeeding within 1 hour after birth.	

#### Essential childbirth-related practices

For a subset of births (approximately 6000), birth attendant behaviors will be directly measured by independent nurse observers to measure the impact of the SCC program on delivery of individual EBPs at the first three of the four junctures: admission, before delivery and within 1 h after delivery (see Table 2). Observation is not made at the time of discharge due to the variety of times at which women are discharged. Data will be collected in intervention and control sites (see Additional file 2 and Additional file 3: Figure S2).

### Data collection

#### Mortality and morbidity primary outcome

Building upon the existing facility-based birth registers in UP, a standardized birth register will be implemented at each study site from which we will extract demographic information relating to the mother and baby; contact information for the family and the family’s community health worker; and in-facility survival outcomes for each mother-baby dyad. Field-based study data collectors will extract the required data and enter the data into a password-protected software application using mobile technology, which will be automatically uploaded to a firewalled, HIPAA-compliant server.

A call center staffed by trained data collectors using standardized scripts will determine outcomes by computer-assisted telephonic interviews (CATI). This tool was developed in consultation with a local communication specialist to best suit the local language, terminology, and sensitivity towards local cultural norms.

The call center staff will attempt to follow up all mother-baby dyads between 8 to 21 days post childbirth to determine patient- or family-reported 0–7-day morbidity and mortality outcomes. Call center staff will call the mother (or if mother is unavailable, the woman’s husband or other immediate family member), reconfirm consent, and, if received, conduct the interview. If contact cannot be completed by telephone, the case will be transferred to field-based data collectors for follow-up through home visit. The field team will visit the home, reconfirm consent and, if received, the field-based data collector will hand a telephone to the woman/family member to go through the CATI interview with call center staff to gather the outcome and patient satisfaction information, or a field worker will collect the data directly if telephone communication cannot be established. Follow-up data will be entered simultaneously into a database using a web-based form integrated with a mobile application.

#### Essential childbirth-related practices

In a subsample of birth events at 30 sites (15 pairs), adherence by health workers to essential practices outlined on the checklist will be assessed starting approximately 8 weeks after the launch of SCC use; additional assessments of EBP will be done 6 and 12 months after the launch. Trained nurse data collectors will directly observe the health workers who attend to women and newborns during their labor and delivery at three of the four checklist pause points. Data were not collected at the fourth checklist pause point due to the variety of times at which women are discharged. Data will be recorded on standardized data collection forms (paper-based) and will immediately then be entered into a mobile application.

#### Facility, safety attitudes, checklist utilization, and patient satisfaction surveys

Three surveys will be implemented at study facilities to understand facility supply availability, health workers’ attitudes about safety and quality of care, and in the intervention sites, health workers’ use of the checklist. In order to obtain data on availability of safe birth supplies, a facility survey will be completed in all 120 facilities at baseline and every 3 months thereafter. The health worker safe attitude surveys will be completed at baseline and every 6 months after the study starts in all 120 sites. Checklist utilization surveys will be completed in the 60 intervention sites at baseline and at 6 and 12 months after intervention initiation.

In the morbidity/mortality outcome interviews of the mother-infant dyads, the interview will finish with patient satisfaction questions, inquiring about satisfaction with delivery experience, understanding of postpartum danger signs, and provision of family planning options.

### Quality control

A robust quality control and monitoring (QCM) component has been developed to maximize the likelihood of quality and consistency in the implementation of the intervention package and assess data quality. The QCM system will have two main components: progress reporting by the implementation teams and in-person monitoring visits by designated supervisors. The coach and coach team leader will record qualitative information and review reports with their supervisors. Team leaders will visit sites regularly to directly observe the quality of intervention implementation, collect data on implementation progress, and measure achievements around (1) rate of Checklist use (compliance), (2) the proportion of cases for which the Checklist was completed, (3) the frequency of coaching visits maintained, (4) the proportion of birth attendants who received training, and (5) skill transfer to the CQCs.

The data quality assurance process has been developed to focus on the accuracy, validity and consistency of research data collected. Supervisors (team leaders) will observe data collectors to assess consistency and accuracy of observation and data recording and proper transfer of data from paper-based to electronic data collection. Further, a call center validation process will be focused on ensuring the validity of call center-collected data compared to home visit-collected information. Finally, the data entry system has built-in quality controls to prevent incorrect data entry (e.g., postpartum complication occurring before admission date) or provide an alert when illogical or out-of-range values are entered.

### Power analysis

The nature of the SCC program as a health facility innovation precludes randomization at the individual patient level; thus, the study is randomized at the cluster (i.e., public sector facility) level. Estimating intracluster correlation coefficients (ICCs) is necessarily based on extrapolations from prior studies. ICC estimates have been derived for maternal and newborn health from the 2005 WHO Global Survey on Maternal and Perinatal Health [26]. Considering these available data, and following discussion with experts in biostatistics, we therefore assume the relatively conservative ICC estimate of ICC = 0.01. Based on available birth delivery load data, we expect the average cluster size to be 1440 births.

Data available from UP suggest that the baseline rate of the primary (combined) outcome may be as high as 120/1000 live births. The baseline rate used in this study will be 60/1000; this is purposively set lower than the estimation due to possible inaccuracies in published data and because available data include community-based birth events, which may have higher mortality rates.

Based on existing health indicators in UP, and the results of the SCC program pilot study that demonstrated marked improvement in health worker performance, it is proposed that the detection of up to a 15.3% reduction in primary outcome with the SCC program is achievable and meaningful in this population. The result (using an ICC = 0.01 and a matching design effect of 0.55) is a trial enrolling 120 facilities (60 pairs of facilities) for a maximum of 171,964 births (approximately 86,000 births in each arm) with 80% power and an alpha value of 0.05. We may have a larger sample size enrolled in order to attain balanced pairs and similar cluster sizes as well as stop the pairs at a similar time point. However, we will have 80% power to detect a smaller reduction (as small as 10%) in the primary outcome, if the matching effect is ultimately stronger than this conservative estimate.

#### Sample size for essential childbirth-related practices (EBPs)

Structured observation of birth attendant performance will be performed on a subsample of births (*n* = 6000) and assess individual behavior observations at each of three SCC pause points (1 observation point for the first 2 pause points, 2 observation points for pause point 3). This will be observed from 15 matched pairs of facilities (30 facilities), or 25% of the total study facilities. For a given EBP, we will measure the outcome at 7–10 weeks of coaching with an ICC = 0.01 and DeffM = 1. To assess sustainability of intervention impact, EBP observations will be repeated at 6 months and 12 months after intervention initiation. Observations will be conducted on individual birth practices (60 births per facility × 30 facilities × 4 observation points), the Rao-Scott chi-square test statistic will have greater than 80% power (with a 5% type I error) to detect a minimum difference in rates between the intervention and control sites of 8.5 per 100 (8.5%).

### Data Safety Monitoring Board

A Data Safety Monitoring Board (DSMB) will be established to oversee the safety and scientific accuracy of the study. The DSMB will contain at least three members external to the study team with at least two members based in India. All members will have familiarity with research in low-resource settings and an understanding of the conditions specific to UP. Membership will include a chairperson and individuals with expertise in biostatistics, obstetrics and neonatal care. The primary responsibilities of the DSMB will be to ensure subject safety and evaluate interim results for possible early stopping (see the next paragraph below).

### Data analysis and dissemination

The Rao-Scott chi-square test statistic, which adjusts for the matched-pair, cluster randomization scheme, will be used to compare the rates of the primary and secondary outcomes in the comparison and intervention groups. The trial will be monitored for possible early stopping if a large intervention effect occurs at the time of interim analysis, using a Haybittle-Peto approach [27]. An interim Rao-Scott chi-square test will be performed and shared with the DSMB after approximately 25% (45,000) of the total births are enrolled. In order to stop the study at this point in favor of the SCC program, the *p* value for this Rao-Scott chi-square test must be ≤0.001. However, the stopping parameters for this study will be used as a guideline rather than as an absolute rule. Any final decision will also consider additional endpoints such as differences in complications, process measures, and birth attendant compliance to the Checklist [17]. If the study is halted by the DSMB, the intervention will be rolled out to all sites.

If the study continues, the final analysis will be after enrollment and follow-up are completed. Using the Haybittle-Peto approach, the *p* value for this Rao-Scott chi-square test must be ≤0.05 for the intervention and comparison groups to be declared significantly different.

Secondary analyses will use the Rao-Scott chi-square test, adjusted for matching and clustering, to compare groups with respect to selected individual health outcomes and birth attendant EBP adherence. Since these are secondary analyses, no formal stopping rules will be applied and all tests will be performed at a 5% type I error rate. The surveys for Checklist utilization, health worker safety attitudes, and patient satisfaction will be analyzed by comparing proportions of responses between the intervention and comparison groups.

The study investigators will have access to the final trial dataset; however, the study biostatistician will have access to the unblinded dataset during the interim analysis. In accordance with the funding agreement with the Bill & Melinda Gates Foundation, the publications will be on “open access” terms and datasets will be accessible. The study results will be disseminated to internal and external collaborators, funders, scientific media, and policymakers, including Government of Uttar Pradesh and Government of India officials. The investigators will take the lead in publication of the study to share results with stakeholders and the broader scientific community.

### Ethical considerations

#### Consent procedures

In order to obtain consent for follow-up at 7 days postpartum of mother-infant pairs, informed verbal consent will be obtained from the mother or her surrogate by health workers when being discharged from study facilities after delivery or at the time of referral. Documentation of verbal consent will be included in the birth register and documented by a witness. A printed information sheet will be provided in Hindi to the mother. When consented mothers receive a follow-up call or home visit, consent will be confirmed.

In the smaller sample of birth events that will be observed for EBP uptake, written informed consent will be obtained from the mother or her surrogate and verbal consent from the health worker conducting the delivery. If the mother or surrogate does not consent, health worker written consent cannot be obtained, or the health worker refuses, the birth event will not be observed.

Participation in the study for the women and health workers will be voluntary with the ability to opt out at any time. No monetary incentives will be provided reflecting the limited time required. In such a case where the mother opts out, the care provided to the mother (and the baby) during the admission and after discharge will not be any different from what she, and the baby, would have received as a study participant.

To protect the privacy of study participants, every birth event will be allotted a unique identifier code; all paper forms will be retained in locked cabinets at the facility and central office. All electronic data will be maintained on a HIPAA-compliant encrypted database.

## Discussion

The Safe Childbirth Checklist is a WHO-branded tool developed by frontline health workers and technical experts in maternal health, newborn health, checklist-based programs, quality improvement, and implementation science [10]. After several years in development, including field-testing for usability and a pilot study of its impact on health worker practices, the multicenter trial, testing the BetterBirth approach to Checklist implementation in UP will now assess the effectiveness of the program in reducing avoidable maternal, fetal, and newborn harm. The trial, as designed, will provide crucial rigorous evidence to the global maternal, neonatal and child health community; this information is anticipated to inform policies and practice towards accelerating the improvement quality and safety of facility-based delivery care and reducing mortality and morbidity of women and newborns. As is the case with many randomized controlled trials in resource-poor settings, the overall intensity of the intervention provided within the trial may be more extensive than what can be brought to scale, but it is structured to allow for the identification of the key elements required for wider-spread success.

### Challenges and opportunities

There are a number of risks to the successful completion of this large, complex trial in UP at each level—state, district, facility, and individual—reflecting local context, knowledge and what is required to successfully implement a Checklist program at scale along with more universal clinical trial challenges. India is a large and diverse country with health agencies at both the national and state levels. Rolling out a health program in the public sector at scale in this context requires collaboration and support from national, state, and district political leaders. To ensure alignment of the trial with policies and priorities at these levels, we will establish regular channels of formal and informal communication and engagement. This includes providing updates on progress and feedback of implementation data, partnering with key figures at each level to hold joint public meetings with national, state, and district leaders to reflect on progress of the program and trial, and providing training to government officials in areas of local interest. These partnerships have ensured that our program and trial design have been carefully reviewed and tailored to ensure alignment with ongoing and planned government programs. This process has also provided key insight regarding the feasibility of study implementation within the infrastructure of UP. Challenges in this area will continue as government officials change positions, highlighting the need to ensure sustained, effective relationships even as personnel change over the relatively long period of data collection.

At all levels, one of the most critical challenges is the need for local champions, as well as strategies for sustainability of the program, particularly given the frequent reassignment of facility staff across UP. To address this potential barrier, we have incorporated the training of a facility-level Childbirth Quality Coordinator (CQC) to continue supporting the use of the SCC during the study and once study coaches have completed the 8-month-long intervention. Further, the engagement process includes a complete discussion of Checklist implementation and data collection. The participation of facility leaders in the engagement and launch processes build local buy-in for Checklist use. Similar processes are held at the district and state levels to ensure that priorities align and health leaders have ownership in the project and dedication to quality of care. All of these processes are designed to sustain the use of the SCC.

Partnerships among the investigators and the use of expert local staff for implementation and management ensure that we remain cognizant of cultural and infrastructure determinants impacting the health care and research environments. These allow us to deal with challenges in ensuring informed consent, with ensuring consistent meaning of questions despite variation in dialects across the state, and with operational issues related to how women use health care facilities and recover postpartum, as well as the role of traditional birth attendants in facility based births.

As has been shown previously, simply adding a checklist to workflow will not create uptake or sustainability [28]. The greatest challenge is in ensuring that individual health workers successfully adopt the SCC program into clinical practice. Sustained behavior change in any health care setting is challenging, especially in resource-limited settings [29]. Early pilot studies resulted in the better understanding of how to educate birth attendants on how to use the SCC and be motivated to improve their own clinical practice [30]. In our lessons learned from the pilot studies, one key finding is the importance of using education and peer-to-peer coaching to empower health workers to believe in their own capacity for progress, and to realize that through their own practices they can ensure that maternal and neonatal outcomes may be improved. Further, with the development of the CQCs and facility staff to take on responsibility and utilization of the SCC, we hope to empower the CQCs such that they will be able to collect, collate, coach, interpret, and feedback data to the frontline workers and provide information to the facility leaders.

The main risk with the data collection stems from the lack of a comprehensive, robust existing health management information system in the study setting. Based on learning from the pilot, the original plan to use standardized birth registers to collect routinely available demographics and in-facility outcome data has required compilation of multiple primary data sources. In baseline data collection, we have successfully collated the data and been able to follow up women and their newborns after discharge by telephone. After the pilot studies, the standard maternal severe morbidity definitions were modified to reflect the available resources. We reviewed a range of methodologies for self-report on these and other time-delineated outcomes [24, 31, 32]. In addition, the use of maternal near-missed deaths as a component of a composite indicator that combines maternal and newborn outcomes is novel. Another potential risk is contamination of the control sites in this matched-pair, randomized controlled trial. However, this is a limited risk as the WHO Safe Childbirth Checklist has been part of the Government of India Maternal Health Toolkit since 2013, yet the Checklist has not been widely adopted.

To mitigate the above risks, we adopted methodological strategies to maximize the implementation of successful trial conduct in this real-world setting. We completed several pilot studies in facilities in UP, using a quality improvement methodology, and then measured success in both effectiveness of education as well as rates of adoption of the care practices comprising the SCC. Through these pilot tests in nine facilities, we progressively modified our approach until we were certain that we had removed all identifiable impediments to successful implementation, data collection, and monitoring [30]. These changes included but were not limited to a complete re-evaluation of the methods for launch and coaching support. Data from this extensive pilot phase will not be used in the final analyses.

In summary, simple, scalable solutions are essential, and desperately needed, to improve maternal and neonatal outcomes around the time of childbirth. In partnership with the co-principal investigators (Ariadne Labs, a joint center of Brigham and Women’s Hospital and Harvard T.H. Chan School of Public Health; Community Empowerment Lab; Jawaharlal Nehru Medical College), Population Services International, the World Health Organization and the Governments of Uttar Pradesh and India, along with guidance by a Scientific Advisory Committee comprised of technical experts in maternal and newborn health, the BetterBirth Trial in UP has been successfully designed and initiated implementation. Utilization of pilot studies and iterative learning has improved the design of the trial and intervention, as well as the data collection systems, for implementation of a high-quality, large-scale study. Studies such as this with large land coverage and sample size require immense coordination at all levels of the health system. If the SCC and coaching intervention are found to reduce maternal, fetal and neonatal harm, patients and other stakeholders stand to benefit from a proven quality improvement strategy that could potentially help influence outcomes in millions of births each year.

## Trial status

The trial has completed planning and pilot phases and is currently enrolling.

## Additional files

Additional file 1: Figure S1. BetterBirth Safe Childbirth Checklist: the WHO Safe Childbirth Checklist adapted for alignment with Indian national guidelines. (PDF 5557 kb)
Additional file 2: BetterBirth trial SPIRIT checklist. (DOC 121 kb)
Additional file 3: Figure S2. BetterBirth trial SPIRIT figure. (DOCX 32 kb)

## Abbreviations

CHC: Community health center
DH: District hospital
DSMB: Data Safety Monitoring Board
EBP: Essential childbirth-related practice
FRU: First Referral Unit
GoI: Government of India
ICC: Intracluster correlation
JNMC: Jawaharlal Nehru Medical College
NHM: National Health Mission
PHC: Primary health center
SCC: Safe Childbirth Checklist
UP: Uttar Pradesh
WHO: World Health Organization
