# Supplementary material for: Effectiveness of the WHO Safe Childbirth Checklist program in reducing severe maternal, fetal, and newborn harm in Uttar Pradesh, India: study protocol for a matched-pair, cluster-randomized controlled trial
Source: Trials. 2016 Dec 7;17:576. doi: 10.1186/s13063-016-1673-x (PMC5142140; doi:10.1186/s13063-016-1673-x)
Supplement: Additional file 1: Figure S1. — BetterBirth Safe Childbirth Checklist: the WHO Safe Childbirth Checklist adapted for alignment with Indian national guidelines. (PDF 5557 kb) [file 13063_2016_1673_MOESM1_ESM.pdf]

# SAFE CHILDBIRTH CHECKLIST

BetterBirth

## Check 1 — On Admission

Instructions:

1. Put the tick ☑ in appropriate box.
2. Follow the information given on right side for reference.

| | |
|---|---|
| Mother's Temperature ____________ <br> Mother's Blood Pressure ____________ | Fetal Heart Rate ____________ |

### Does Mother need referral?

- ☐ No
- ☐ Yes, organized

Refer to FRU if any of following danger signs are present and state reason on transfer note:

- Vaginal bleeding
- High fever
- Severe headache or blurred vision
- Convulsions
- Severe abdominal pain
- History of heart disease or other major illnesses
- Difficulty in breathing

### Partograph started?

- ☐ No, will start at ≥4cm
- ☐ Yes

Start when cervix ≥4 cm,

- Every 30 min: plot contractions, FHS, and maternal pulse
- Every 4 hours: plot temperature, blood pressure, and cervical dilation in cm (cervix dilates ≥ 1 cm/hr)

### Does Mother need:

*Antibiotics?*

- ☐ No
- ☐ Yes, given

Give antibiotics to Mother if:

- Mother's temperature >38$^0$C (>100.4$^0$F)
- Foul-smelling vaginal discharge
- Rupture of membranes >12 hrs without labor or >18 hrs with labor
- Labor >24 hrs or obstructed labor
- Rupture of membranes <37 wks gestation

*Magnesium sulfate?*

- ☐ No
- ☐ Yes, given

Give first dose and then refer immediately to FRU if Mother has:

- If diastolic BP is ≥ 110 mm Hg and 3+ proteinuria
- Convulsions

### What is HIV Status of Mother?

- ☐ Positive
- ☐ Negative
- ☐ Status unknown, HIV test advised

If mother is HIV positive :

- Give Nevirapine
- If not available, refer the patient immediately after birth

### Are soap, water and gloves available?

- ☐ No
- ☐ Yes, I will wash hands and wear gloves for each vaginal exam

☐ **Presence of Birth Companion at birth encouraged.**

☐ **Confirmed that Mother or Companion will call for help during labor if needed**

Call for help if any of:

- Bleeding
- Severe abdominal pain
- Difficulty in breathing
- Severe headache or blurred vision
- Urge to push
- Cannot empty bladder frequently

Patient Name ............................................
Registration No. ...........................................

BetterBirth

Completed by ................................. Date .....................

# Check 2 — Just Before Pushing (or Before Caesarean)

| | |
|---|---|
| Mother's Temperature ____________<br>Mother's Blood Pressure____________ | Fetal Heart Rate________________ |

## Does Mother need:

*Antibiotics?*

☐ No
☐ Yes, given

Give antibiotics to Mother if any of:
- Mother's temperature >38$^0$C (>100.4$^0$F)
- Foul-smelling vaginal discharge
- Rupture of membranes >18 hrs with labor
- Labor >24 hrs or obstructed labor now
- Cesarean section

*Magnesium sulfate?*

☐ No
☐ Yes, given

Give first dose and then refer immediately to FRU if Mother has:
- If diastolic BP is ≥ 110 mm Hg and 3+ proteinuria
- Convulsions

## Confirm essential supplies are at bedside:

*For Mother*

☐ Gloves
☐ Soap and clean water
☐ Oxytocin 10 units in syringe
☐ Pads for Mother

Prepare to care for Mother immediately after birth
1. Confirm single baby only (not multiple birth)
2. Give oxytocin IM within 1 minute
3. Massage uterus after placenta is delivered
4. Confirm uterus is contracted

*For Baby*

☐ Clean towel
☐ Sterile scissors / blade to cut cord
☐ Cord ligature
☐ Mucus extractor
☐ Bag-and-mask

Prepare to care for Baby immediately after birth
1. Keep the baby dry and warm, give IM Vit. K
2. If not breathing: stimulate and clear airway
3. If still not breathing:
   a. Clamp and cut the cord
   b. Ventilate with bag-and-mask
   c. Shout for help (Pediatrician/LMO/CCSP trained service provider

☐ **Assistant identified and ready to help at birth if needed.**



Completed by .................................  Date .....................

## Check 3 — Soon After Birth (within 1 hour)

| Mother | Baby |
|---|---|
| Mother's Temperature ________ | Baby's Temperature ________ |
| Mother's Blood Pressure ________ | Baby's Respiratory Rate ________ |
| | Baby's Weight ________ |

### Is Mother bleeding abnormally?

☐ No

☐ Yes, shout for help

If bleeding >500 ml, or 1 pad soaked in <5 min:

- Massage uterus
- Start I/V fluids
- Treat cause
- If placenta delivered or completely retained: give IM or I/V Oxytocin, stabilize, and refer to FRU
- If placenta is incomplete: remove if any visible pieces, and refer immediately to FRU

### Does Mother need:

*Antibiotics?*

☐ No

☐ Yes, given

Give antibiotics to Mother if manual removal of placenta performed, or if Mother's temperature >38$^0$C (>100.4$^0$F) and any of:

- Chills
- Foul-smelling vaginal discharge

*Magnesium Sulfate?*

☐ No

☐ Yes, given

Give first dose and then refer immediately to FRU if Mother has:

- If diastolic BP is ≥ 110 mm Hg and 3+ proteinuria
- Convulsions

### Does Baby need:

*Antibiotics?*

☐ No

☐ Yes, given

Give Baby antibiotics if antibiotics were given to Mother, or if Baby has any of:

- Breathing too fast (>60/min) or too slow (<30/min)
- Chest in-drawing, grunting, or convulsions
- Looks sick (lethargic or irritable)
- Too cold (Baby's temp <35$^0$C and not rising after warming) or too hot (Baby's temp>38$^0$C)

*Referral?*

☐ No

☐ Yes, organized

Refer Baby to FRU if:

- Any of the above (antibiotics indications)
- Baby looks yellow, pale or bluish

*Special care and monitoring?*

☐ No

☐ Yes, organized

Arrange special care / monitoring for Baby if any of:

- Preterm
- Birth weight <2500 gms
- Needs antibiotics
- Required resuscitation

*Nevirapine?*

☐ No

☐ Yes, given

If Mother is HIV+, follow local guidelines for baby (prophylaxis to be started within 12 hours after birth)

☐ **Started breastfeeding and skin-to-skin contact (if Mother and Baby are well). Importance of colostrum feeding explained.**

☐ **Danger signs explained and confirmed that Mother/Companion will call for help if danger signs appear. (Refer to "Danger Signs" given under Check 4).**



Completed by ................................ Date ....................

## Check 4 — Before Discharge

| Mother's Temperature ________ | Baby's Temperature ________ |
|---|---|
| Mother's Blood Pressure ______ | Baby's Respiratory Rate ______ |

### Is Mother's bleeding controlled?

- ☐ No: Treat, observe and refer to FRU if needed
- ☐ Yes

### Does Mother need antibiotics?

- ☐ No
- ☐ Yes: Give antibiotics and delay discharge

Give antibiotics to Mother if her temperature >$38^0$C or >$100.4^0$F and any of:

- Chills
- Foul-smelling vaginal discharge

### Does Baby need antibiotics?

- ☐ No
- ☐ Yes: Give antibiotics, delay discharge, and refer to FRU

Give Baby antibiotics if Baby has any of:

- Breathing too fast (>60/min) or too slow (<30/min)
- Chest in-drawing, grunting, or convulsions
- No movement on stimulation
- Too cold (Baby's temp <$35^0$Cand not rising after warming) or too hot (Baby's temp >$38^0$C)
- Stopped breastfeeding well
- Umbilicus redness extending to skin or pus discharge

### Is Baby feeding well?

- ☐ No: Help in baby feeding, delay discharge and refer to FRU if needed
- ☐ Yes, teach Mother exclusive breastfeeding

**☐ Home transport and follow-up for Mother and Baby arranged.**

**☐ Confirmed that BCG and Polio first dose given to Baby.**

**☐ Family planning options discussed and offered to Mother:**

- LAM
- IUCD
- Female Sterilization
- OCP
- Condoms
- Male Sterilization

**☐ Danger signs explained and confirmed that Mother/Companion will seek help, if danger signs appear after discharge.**

## Danger Signs

***Mother** has any of:*

- Bleeding
- Severe abdominal pain
- Severe headache or blurred vision
- Breathing difficulty
- Fever or chills
- Difficulty in emptying bladder

***Baby** has any of:*

- Fast / difficulty breathing
- Fever
- Unusually cold
- Stops feeding well
- Less activity than normal
- Whole body becomes yellow

Based on the WHO Safe Childbirth Checklist.
All reasonable precautions have been taken to verify the information contained in this publication. The responsibility for the interpretation and use of the materials lies with the reader.

BetterBirth

Completed by ................................. Date .....................
