# Supplementary material for: Effectiveness of the WHO Safe Childbirth Checklist program in reducing severe maternal, fetal, and newborn harm in Uttar Pradesh, India: study protocol for a matched-pair, cluster-randomized controlled trial
Source: Trials. 2016 Dec 7;17:576. doi: 10.1186/s13063-016-1673-x (PMC5142140; doi:10.1186/s13063-016-1673-x)
Supplement: Additional file 3: Figure S2. — BetterBirth trial SPIRIT figure. (DOCX 32 kb) [file 13063_2016_1673_MOESM3_ESM.docx]

|  | Facility Enrollment | |  | | | | | | | | | | | | | |
| --- | --- | --- | --- | --- | --- | --- | --- | --- | --- | --- | --- | --- | --- | --- | --- | --- |
|  | District Engage | Facility Engage & Launch | Individual Participant Enrollment | | | | | | | | | | | | | |
| TIMEPOINT (Weeks)* | 0 | 4 | 8 | 12 | 16 | 20 | 24 | 28 | 32 | 36 | 40 | 44 | 48 | 52 | 56 | 60 |
| Enrollment: |  | | | | | | | | | | | | | | | |
| District Chief Medical Officer Approval | X |  |  |  |  |  |  |  |  |  |  |  |  |  |  |  |
| Head of Facility Approval |  | X |  |  |  |  |  |  |  |  |  |  |  |  |  |  |
| Facility Participant List |  | X |  |  |  |  |  |  |  |  |  |  |  |  |  |  |
| Patient Consent for follow-up |  |  |  |  |  |  |  |  |  |  |  |  |  |  |  |  |
| Patient Consent for Observation** |  |  |  |  |  |  |  |  |  |  |  |  |  |  |  |  |
| Healthcare Worker Consent for Observation** |  |  |  |  |  |  |  |  |  |  |  |  |  |  |  |  |
| Intervention: |  |  |  |  |  |  |  |  |  |  |  |  |  |  |  |  |
| BetterBirth Program |  |  |  |  |  |  |  |  |  |  |  |  |  |  |  |  |
| Assessments: |  | | | | | | | | | | | | | | | |
| Safe Birth Registry |  |  |  |  |  |  |  |  |  |  |  |  |  |  |  |  |
| Computer Assisted Telephonic Interview |  |  |  |  |  |  |  |  |  |  |  |  |  |  |  |  |
| Essential Birth Practices Observation |  |  |  |  |  |  |  |  |  |  |  |  |  |  |  |  |
| Health Worker Safe Attitudes Survey |  | X |  |  |  |  |  | X |  |  |  |  |  | X |  |  |
| Checklist Utilization Survey |  |  | X |  |  |  |  |  | X |  |  |  |  |  | X |  |
| Facility Survey |  | X |  |  | X |  |  | X |  |  | X |  |  | X |  |  |
| Patient Satisfaction Survey |  |  |  |  |  |  |  |  |  |  |  |  |  |  |  |  |

**Additional file 3: Figure S2.** BetterBirth Trial SPIRIT Figure.

*Matching and assignment of intervention and control occurred before the start of any study activity in any facility

**Data collection of essential birth practices is collected on a subset of 30 sites (15 intervention-control pairs)
